# Influence of gender and combined estrogen–progestin oral contraceptive on parotid saliva flow rate, pH, and electrolytes concentration

**DOI:** 10.1002/cre2.800

**Published:** 2023-11-07

**Authors:** Angela Rovera, Paul Anderson

**Affiliations:** ^1^ Dental Physical Sciences Unit, Centre for Oral Bioengineering, Institute of Dentistry Queen Mary University of London London UK

**Keywords:** diagnostics, oral contraceptive, parotid gland, saliva

## Abstract

**Objectives:**

Endocrinal variations within an individual impact the electrolyte composition, pH, and flow‐rate (FR) of saliva. The aim of this study was to evaluate the gender‐specific differences and the effect of combined estrogen‐progestin oral contraceptives (COCs) on FR, pH, and electrolyte concentrations in the parotid saliva (PS) of a group of healthy adults.

**Material and Methods:**

Stimulated PS was collected from 20 healthy adults using a Lashley cup; 11 males, 8 females, and 1 female undertaking combined contraceptive therapy (levonorgestrel/ethinyloestradiol 0.1 mg + 0.02 mg). FR and pH were recorded for each saliva sample. Electrolytes concentrations (Na^+^, Ca^2+^, K^+^, Mg^2+^) were measured using inductively coupled plasma optical emission spectrometer (ICP‐OES). Statistical analysis was performed, and the significance level was set at *p* < .05.

**Results:**

PS FR varied from 0.13 to 0.42 mL/min in females not taking any medication and from 0.08 to 0.5 mL/min in males not taking any medication. PS pH of females and males not taking any medication ranged from 6.23 to 7.50 and from 6.15 to 7.55. PS pH and FR of the female taking COCs were 6.5 and 0.1 mL/min. PS pH, FR, and electrolytes concentrations (Ca^2+^, Na^+^, K^+^, Mg^2+^) were not statistically significantly different between females and males not taking any medication. PS concentrations of Ca^2+^ and Na^+^ were significantly higher in the females taking COCs than in the females not taking any medication. Whereas, concentrations of K^+^ and Mg^2+^ did not differ significantly between the females taking COCs and the females not taking any medication.

**Conclusions:**

There are no significant gender‐specific differences in PS flow rate, pH, and electrolyte concentrations of Na^+^, Ca^2+^, Mg^2+^, and K^+^. Combined hormonal oral contraceptive has a significant effect on PS flow rate, pH, Ca^2+^, and Na^+^ concentrations. Whereas the PS concentration of K^+^ and Mg^2+^ are not influenced by COCs. These results warrant further investigation.

## 1 INTRODUCTION

The endocrinal variations of an individual impact the electrolytes composition, pH, and flow rate (FR) of saliva (Mandel, 1993). Despite that estrogen hormones have been reported to influence saliva secretion (Percival et al., 1994), specific gender differences in parotid saliva (PS) electrolytes composition, pH, and FR have not been consistently identified.

Half the volume of stimulated saliva is secreted from the parotid glands which can be canulated to collect sterile saliva (Azevedo et al., 2008).

Several reports have shown that females have a significantly lower mean FR than males for whole saliva and for PS (Heintze et al., 1983; Percival et al., 1994). However, many other studies did not find significant gender differences in FR (Ben‐Aryeh et al., 1984; Heft & Baum, 1984). Further, the specific gender differences in salivary pH values remain controversial. Several reports have shown that the pH value of saliva does not differ, or only slightly differs between genders (Bel'skaya et al., 2020; Ben‐Aryeh et al., 1984). However, specific gender differences in saliva pH values have been seen in several other studies reporting lower salivary pH values in females than males (Li‐Hui et al., 2016; Prodan et al., 2015).

Despite the fact that many studies have indicated that hormones influence the electrolyte composition of female saliva, particularly at the time of ovulation (Laine et al., 1991), no significant changes in calcium, sodium, or potassium ion concentrations in PS during the menstrual cycle (Puskulian, 1972). However, ovulation is prevented by combined estrogen–progestin oral contraceptives (COCs) (Laine et al., 1991), which contain synthetic estrogens (i.e., ethinyloestradiol) and progestins (i.e., levonorgestrel). The influence of these COCs on PS electrolyte composition has rarely been studied directly; only the study by Magnusson et al. (1975) reported increased sodium and hydrogen ion concentrations during the use of COCs (Magnusson et al., 1975). The hormone preparation used in their study was norgestrel 0.5 mg + ethinyloestradiole 0.05 mg. The first COCs available contained a high dose of hormones and had high rates of cardiovascular complications and undesirable side effects (Gallo et al., 2013). Thus, the changes in COCs formulations over time have increased safety and decreased side effects (Gallo et al., 2013).

Because the use of saliva as a diagnostic marker has become more widespread in clinical chemistry in recent years (Mandel, 1990), and COCs are the most common form of reversible hormonal contraception (Vibarel‐Rebot et al., 2015), it is important to understand the influence of gender and use of COCs on saliva flow rate, pH, and electrolytes concentration. Therefore, the aim of this study was to evaluate the gender‐specific differences and the combined estrogen‐progestin contraceptive therapy effects on FR, pH, and electrolyte concentrations (Na^+^, Ca^2+^, K^+^, Mg^2+^) in the PS of a group of healthy adults.

## 2 MATERIALS AND METHODS

The study was conducted in accordance with the Declaration of Helsinki and approved by the Ethics Committee of ASO Santa Croce e Carle Cuneo, Italy (n.66‐17 of May 5, 2017).

### 2.1 Sample collection and preparation

PS samples were collected by the same dentist from 20 subjects; 11 males and 8 females, with 1 female undertaking combined contraceptive therapy (levonorgestrel/ethinyloestradiol 0.1 mg + 0.02 mg). The sample population age ranged between 27 and 38 years. PS samples were collected from subjects with a body mass index between 18 and 28.

The exclusion criteria comprised smokers, pregnant women, current users of any regular medication or therapy except for COCs, dry mouth symptoms, and acute illness within the 2 weeks preceding the start of the study. In addition, subjects were excluded from the study if they presented with clinically significant abnormalities in clinical chemistry or hematology, or if there was evidence of a risk of transmitting the agents responsible for acquired immune deficiency syndrome, hepatitis B or C. Further, excessive intake of alcohol, defined as regular maximum weekly intake of greater than 28 units, was also used to exclude subjects from the study (Sreebny & Schwartz, 1986). The subjects were asked not to eat or drink except water nor perform oral hygiene practices and not to undergo heavy physical stress, at least 2 h before saliva collection.

Unilateral chew‐stimulated PS sample collection was performed using a Lashley cup placed over the Stenson's duct (Lashley, 1916). PS samples were collected from each subject into sterilized low‐affinity conical plastic collection tubes (Fisher Scientific) under 1.0 mL of paraffin oil to avoid CO_2_ loss (Bardow, Moe, et al., 2000). The time of collection was fixed between 9 and 11 a.m. The duration of saliva collection was recorded to calculate the FR (mL/min). All saliva tubes were kept on ice from the moment of collection and immediately frozen (−35°C) for 3 days (Chiappin et al., 2007). Subsequently, the PS samples were thawed and centrifuged immediately before pH and inductively coupled plasma optical emission spectroscopy (ICP‐OES) analysis; both were carried out on the same day. Recent findings (Gardner et al., 2018) indicate that saliva components are resilient to freezing. Thawed PS shows only a very little precipitate (compared to submandibular and sublingual saliva) suggesting that this was not important as the object of the study is not the protein component (Francis et al., 2000).

### 2.2 pH measurement

The pH of the PS was measured at room temperature using a digital pH meter (pH 11 Series, Oakton Instruments). The default temperature was set at 25.0°C for each measurement. The pH meter was calibrated using freshly prepared buffers of pH 4.0, pH 7.0, and pH 10.0. The pH meter tip was rewashed with deionized water and dried before each measurement. All pH measurements were carried out in sterilized low‐affinity conical plastic collection tubes (Fisher Scientific), identical to those used for the sample collection, each containing 5 mL of PS.

### 2.3 ICP‐OES sample preparation and analysis

The ICP‐OES analysis was used to detect K^+^, Na^+^, Ca^2+^, and Mg^2+^ in PS. Each metal ion was detected and quantified using ICP‐OES (ICP; Varian Vista‐PRO, Varian Ltd.). Each of the 20 PS samples was processed by acid digestion: 1 mL of PS, 2% HNO_3_, and 8.8 mL of pure deionized water.

The accuracy of the ICP‐OES analytical protocol was evaluated via analysis of certified reference standard materials (Tatro & Amarasiriwardena, 2016).

### 2.4 Statistical analysis

All data were recorded and statistically analyzed using Microsoft Excel 2011 (Microsoft Corporation) and SPSS Statistics (IBM). The sample size was determined based on previous studies (Magnusson et al., 1975; Rovera et al., 2020). Distribution analysis of data normality was assessed using the Shapiro–Wilk test (normality *p* > .05).

Single‐sample two‐tailed *t* tests were applied to compare the group mean to a single value when variables were normally distributed. Specifically, single‐sample two‐tailed *t* tests were used to determine if the values of pH, FR, and ion concentrations (K^+^, Ca^2+^) in the PS of the females not taking any medication were significantly different from the values of the females taking COCs. The significance level was set at *p* < .05.

A one‐sample Wilcoxon signed‐rank test was applied to determine whether the median of a sample was equal to a value when variables were not normally distributed. Specifically, one‐sample Wilcoxon signed‐rank test was used to determine if the values of PS ions concentrations (Na^+^, Mg^2+^) of females not taking any medication were significantly different from values of females taking COCs. The significance level was set at *p* < .05.

Independent sample *t* tests were applied to compare the means of two independent groups to determine whether there is statistical evidence that means are significantly different, when variables were normally distributed. Specifically, independent sample *t* tests were used to compare values of pH, FR, and ions concentration (K^+^, Ca^2+^) in the PS of females not taking any medication to values of males not taking any medication. The significance level was set at *p* < .05.

A Mann–Whitney *U* test was applied to compare variables from two independent groups when the variables were not normally distributed. Specifically, Mann–Whitney *U* tests were used to compare values of ion concentrations (Na^+^, Mg^2+^) of PS from females not taking any medication, which were significantly different from values of PS ion concentrations from males not taking any medication. The significance level was set at *p* < .05.

## 3 RESULTS

Table 1 shows the pH, FR, and ions concentration (ppm) in the PS of the one female taking combined estrogen–progestin oral contraceptives (F‐COCs), the eight females not taking any medication (F), and the eleven males (M) not taking any medication.

**Table 1:** Subjects' gender, PS ion concentrations (ppm), pH, and flow rate (mL/min).

Subject no.	Gender	pH	Flow rate	K^+^	Na^+^	Ca^2+^	Mg^2+^
1	F‐COCs	6.5	0.1	54.12	25.33	6.48	0.08
2	F	6.84	0.23	46.00	8.67	5.47	0.10
3	F	7.5	0.28	49.24	12.14	8.70	0.09
4	F	6.99	0.31	18.41	12.75	4.26	0.10
5	F	7.49	0.36	34.79	5.79	3.66	0.24
6	F	6.85	0.31	73.55	1.68	6.77	0.27
7	F	7.43	0.25	49.66	3.05	2.67	0.08
8	F	7.35	0.42	11.37	0.20	1.01	0.03
9	F	6.23	0.13	39.85	8.75	2.63	0.08
Mean		7.02	0.29	40.36	6.63	4.40	0.12
Median		7.17	0.30	42.93	7.23	3.96	0.10
10	M	6.41	0.08	41.59	9.32	4.87	0.09
11	M	7.52	0.25	43.75	7.82	6.29	0.14
12	M	6.38	0.1	24.48	8.52	3.80	0.09
13	M	7.17	0.25	55.02	1.61	4.21	0.19
14	M	7.6	0.38	65.25	17.32	5.44	0.28
15	M	6.57	0.14	58.55	9.64	5.16	0.05
16	M	7.55	0.56	52.14	2.48	5.15	0.12
17	M	6.99	0.17	47.99	3.99	5.54	0.13
18	M	7.53	0.24	45.44	1.75	3.32	0.15
19	M	6.15	0.11	20.67	0.65	1.11	0.05
20	M	7.09	0.14	52.67	4.25	8.55	0.06
Mean		7.00	0.22	46.14	6.12	4.86	0.12
Median		7.09	0.17	47.99	4.25	5.15	0.12

*Note*: Data are ordered according to gender.

Abbreviations: F, female; F‐COCs, female undertaking estrogen–progestin oral contraceptive (COCs) (levonorgestrel + ethinyloestradiol: 0.1 mg + 0.02 mg); M, male; PS, parotid saliva.

### 3.1 PS pH and flow rate

**Figure 1:**
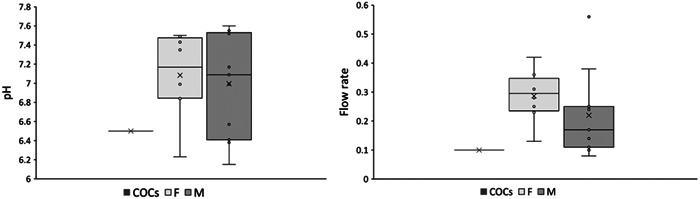
Box and whisker plots of parotid saliva (PS) pH and flow rate (mL/min) values of females taking COCs, females and males not taking any medication.

Figure 1 shows the distribution of pH and FR values in the PS of the 1 female taking COCs and for the 8 females and the 11 males not taking any medication. Data are normally distributed for pH and FR (Shapiro–Wilk test *p* > .05).

The pH of the PS samples of females not taking any medication ranged from 6.23 to 7.50 with a median value of 7.1. The pH of the PS samples of males not taking any medication ranged from 6.15 to 7.55 with a median value of 7.09.

The PS FR in females not taking any medication varied from 0.13 to 0.42 mL/min with a median value of 0.30 mL/min. The FR in the PS samples in males not taking any medication ranged from 0.08 to 0.56 mL/min with a median value of 0.17 mL/min.

The independent samples *t* test two‐tailed results in Table 2 show there was no statistically significant difference in both the pH and FR of the PS comparing females and males not taking any medication (*p* > .05).

**Table 2:** Independent samples *t* test two‐tailed comparing values of PS pH, flow rate (mL/min) of females and males not taking any medication.

	*t* Value	*p* Value
pH	0.38	.71
Flow rate	1.15	.26

Abbreviation: PS, parotid saliva.

Table 3 shows single‐sample two‐tailed *t* tests results comparing values of pH and FR of the PS of females taking COCs to values of females not taking any medication. The pH of the PS of the female taking COCs is significantly lower than that of the females not taking any medication (*p* < .05). The FR of PS of the female taking COCs is also significantly lower than the FR of females not taking any medication (*p* < .05).

**Table 3:** Single‐sample *t* test two‐tailed comparing PS pH, flow rate (mL/min), and ions concentration (ppm) values of females taking combined estrogen–progestin oral contraceptives (COCs) to values of females not taking any medication.

	*t* Value	*p* Value
pH	3.72	.0074*
Flow rate	6.03	.0005*

Abbreviation: PS, parotid saliva.

* Statistical significance at the .05 level.

### 3.2 PS electrolytes

**Figure 2:**
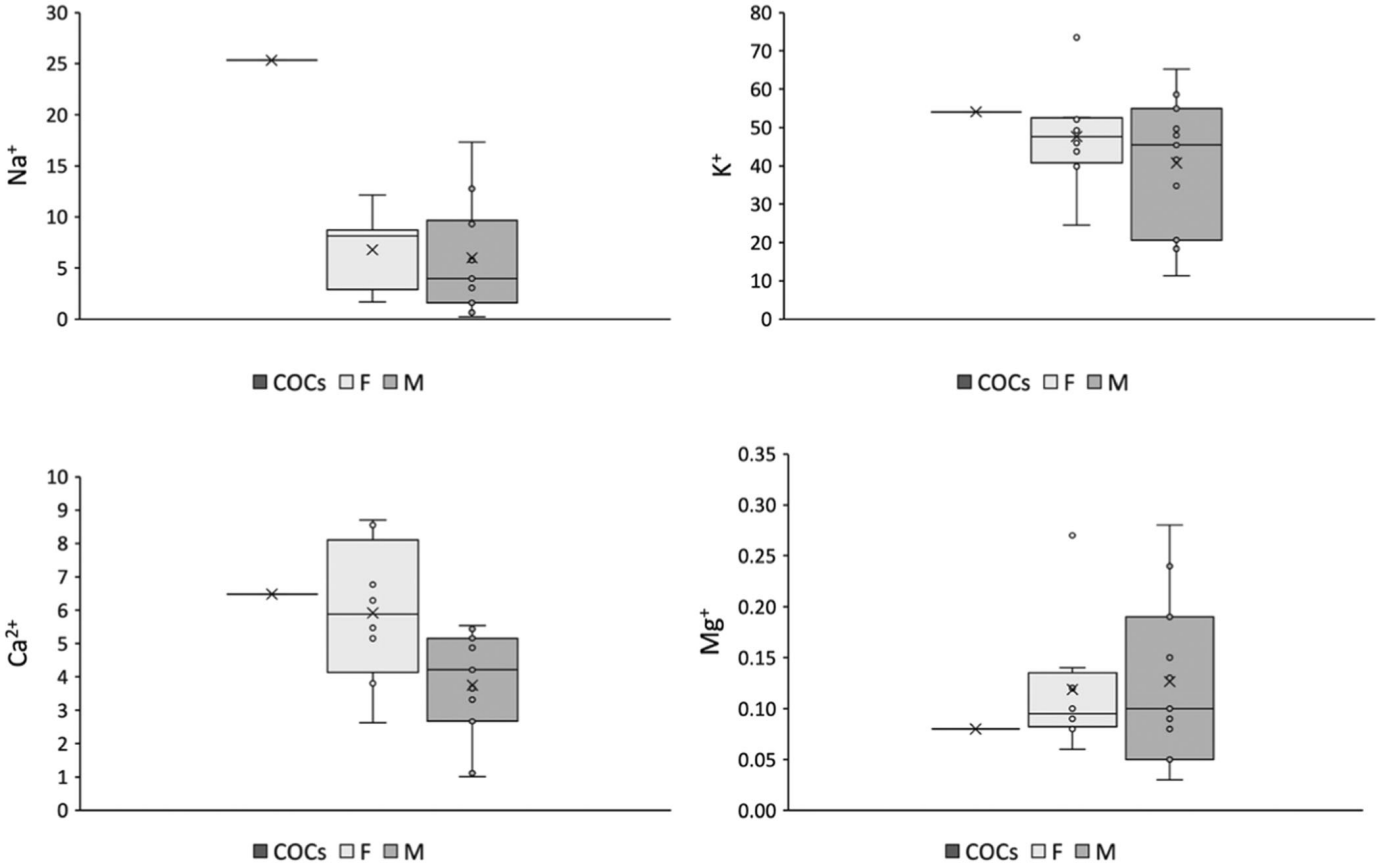
Box and Whiskers of parotid saliva (PS) ions concentrations (ppm) in females taking COCs, females and males not taking any medication.

Figure 2 shows the distribution of metal ion concentrations (Ca^2+^, Na^+^, K^+^, Mg^2+^) in the PS of the female taking COCs, and for the females and males not taking any medication showing the wide variation between subjects. The K^+^ and Ca^2+^ concentrations show an almost normal distribution between subjects (Shapiro–Wilk test *p* > .05). Whereas, the Na^+^ and Mg^2+^ concentrations are not normally distributed (Shapiro–Wilk test *p* < .05).

Table 4 shows that none of the metal ion concentrations (Ca^2+^, Na^+^, K^+^, Mg^2+^) are statistically significantly different between females and males not taking any medication (*p* > .05).

**Table 4:** Independent samples *t* test two‐tailed and Mann–Whitney test comparing values of PS ions concentrations (ppm) of females and males not taking any medication.

	Statistical test	Value	*p* Value
K^+^	Independent samples *t* test two‐tailed	−0.76	.45
Ca^2+^	Independent samples *t* test two‐tailed	−0.46	.64
Na^+^	Mann–Whitney test	−0.28	.77
Mg^2+^	Mann–Whitney test	0.29	.77

Abbreviation: PS, parotid saliva.

Table 5 shows that for the metal ion concentrations of the PS, the Ca^2+^and the Na^+^ concentrations were significantly higher in the female taking COCs than in the females not taking any medication (*p* < .05). Whereas, the K^+^ and Mg^2+^ concentrations did not differ significantly between the female taking COCs and females not taking any medication (*p* > .05).

**Table 5:** Single‐sample *t* test two‐tailed and one‐sample Wilcoxon signed‐rank test comparing PS ions concentration (ppm) values of females taking combined estrogen–progestin oral contraceptives (COCs) to values of females not taking any medication.

	Statistical test	Value	*p* Value
K^+^	Single‐sample *t* test two‐tailed	−1.99	.085
Ca^2+^	Single‐sample *t* test two‐tailed	−2.36	.049*
Na^+^	One‐sample Wilcoxon signed‐rank test	−2.52	.01*
Mg^2+^	One‐sample Wilcoxon signed‐rank test	1.37	.17

* Statistical significance at the .05 level.

## 4 DISCUSSION

The present study results show that there are no significant gender differences in the FR, pH, and electrolyte composition of PS. However, this study *does* show a significant hormonal influence of COCs on the FR, pH, Ca^2+^, and Na^+^ concentration in PS, whereas the concentrations of K^+^ and Mg^2+^ are not influenced.

### 4.1 Flow rate

This study has confirmed that there are no gender differences in the FR of PS in humans. Thus, the endogenous steroid hormones produced during the menstrual cycles do not account for variation in the FR of PS in healthy young females compared to males. Body profile differences in healthy humans may explain the FR gender differences reported by previous studies (Heintze et al., 1986; Percival et al., 1994), since the stimulated FR is directly related to gland size and body size (Dawes et al., 1978; Ericson, 1971; Inoue et al., 2006).

The present study has shown that the FR of PS of the female taking COCs was significantly lower than the FR of females not taking any medication (*p* < .05). This could be explained by the influence of estrogen and progesterone on body fluid regulation that, in turn, can lead to significant fluid retention (Stachenfeld et al., 1999). Previous studies have demonstrated that the osmotic thirst and arginine vasopressin responses to hypertonicity, occur with elevations in estrogen and progesterone, such as during the luteal phase of the menstrual cycle (Spruce et al., 1985; Vokes et al., 1988) and during pregnancy (Davison et al., 1984).

### 4.2 pH

This study has shown that there are no gender differences in the pH values of PS. This is because the concentration of HCo_3_^−^ is strongly dependent on the flow rate (Bardow, Madsen, et al., 2000), which in the present study has not shown to differ between genders. The present study results are in contrast with the findings of Prodan et al. (2015) who reported that salivary pH distribution in females is significantly shifted toward a more acidic pH compared with that of males (Prodan et al., 2015). The observed gender‐related biochemical differences in saliva may be a result of different factors, such as the influence of anxiety (Said et al., 2020), different gene expression profiles (Srivastava et al., 2008), and gland size (Inoue et al., 2006).

The results reported here show the significant influence of COCs in lowering the pH of PS. This is explained by the reduction in the FR of PS in females taking COCs which, in turn, influences the saliva pH. The recent identification of estrogen receptors ß (ER ß) in the salivary gland acinar and ductal cells confirms that estrogens regulate the physiology of these tissues influencing saliva secretion and composition (Valimaa et al., 2004). The evidence that COCs lower the saliva pH should be considered for dental caries risk assessment and prevention.

### 4.3 Electrolytes

This study has shown that there are no significant differences in the metal ion concentration of PS electrolytes between genders. In the literature, significant differences in saliva composition have been found when male patients were compared to female patients with ongoing psychological stress, whereas such differences were not present when healthy populations were investigated (Somer et al., 1993).

As previously reported by Magnusson et al. (1975), the present study has shown a significant increase in the concentration of Na^+^ in PS of females taking COCs compared to females not taking any medication (Magnusson et al., 1975). This may be explained by the fact that COCs increase the renal tubular responsiveness to changes in sodium intake, and sodium retention is a potential mechanism whereby COCs could lead to an increase in blood pressure (Pechère‐Bertschi et al., 2003). The evidence that PS reflects the Na^+^ changes consequent to hormonal contraception should be considered for the follow‐up of patients to prevent hypertension.

Salivary acinar cells are salt‐secreting, and it is the movement of salt across the acinar epithelium from tissue fluid into acinar lumena that leads to water movement and the formation of salivary fluid (Proctor, 2016). It is possible for salt to move across acinar cells because of the activity of the sodium/potassium ATPase (sodium pump), located in the basolateral membrane of acinar cells, which maintains a low intracellular sodium concentration relative to the extracellular tissue fluid (Proctor, 2016). Under physiological conditions, the difference in sodium concentration, the sodium gradient, provides the impetus for the movement of ions (principally sodium and chloride) (Proctor, 2016). However, the exogenous/synthetic hormones, COCs, alter the normal endocrinal system/balance and exert a direct effect on the renin–angiotensin–aldosterone system by altering water and electrolyte transport by the gland. The study of McKinley et al. (1979) suggests a direct effect of angiotensin II at the parotid gland, possibly by a constrictor action on its vasculature or by altering water and electrolyte transport by the gland causing a greater reduction in saliva secretion rate (McKinley et al., 1979). Furthermore, a previous study by Kraintz et al. (1972) reports that aldosterone acts on Na^+^–K^+^ exchange sites in the ducts (Kraintz et al., 1972). Indeed, the regulation of the endocrinal system outside the physiological balance after COCs intake involves a multifactorial process affecting different mechanisms of regulation and salivary gland secretion.

The explanation for the significant increases in the concentration of Ca^2+^ found between the females taking COCs and females not taking any medication may be similar to that reported for the impact of similar drugs in serum. Hartard et al. (1997) reported elevations of Ca^2+^ in the serum of 138 women (aged between 20 and 35 years) (Hartard et al., 1997). They explained that endogenous hypophyseal‐releasing hormones, estrogen, and progesterone peaks are suppressed by low‐dosed oral contraceptives (OCs), and that the use of low‐dosed OCs might counteract the beneficial influence of physical activity on bone formation.

### 4.4 Strength and limitation

The limitation of this study is the small sample size which may affect the generalization of the results. Further, saliva‐based studies are needed to confirm this study's preliminary results.

The strength of the study is that the samples were collected directly from the PS gland, and there was little possibility for the samples to include microbial and other host products, which may confound subsequent analysis.

## 5 CONCLUSIONS

This study shows that there are no significant gender‐related differences in the flow rate, pH, and electrolyte concentrations of sodium, calcium, potassium, and magnesium ions in the PS of healthy young adults.

However, this study does show that the combined estrogen–progestin oral contraceptives do exert a significant effect on PS decreasing the flow rate, decreasing the pH, and increasing the concentrations of calcium (Ca^2+^) and sodium (Na^+^) but not the concentration of either potassium (K^+^) or magnesium (Mg^2+^) ions.

Future research on a large sample size could provide more definitive evidence.

## AUTHOR CONTRIBUTIONS

**Angela Rovera**: Conceptualization; methodology; validation; formal analysis; investigation; resources; data curation; writing—original draft preparation; writing—review and editing; visualization; project administration. **Paul Anderson**: Validation; resources; writing—original draft preparation; writing—review and editing; visualization; supervision; project administration.

## CONFLICT OF INTEREST STATEMENT

The authors declare no conflict of interest.

## ETHICS STATEMENT

Informed consent was obtained from all subjects involved in the study. Written informed consent has been obtained from the patients to publish this paper. The study was conducted in accordance with the Declaration of Helsinki and approved by the Ethics Committee of ASO Santa Croce e Carle Cuneo, Italy (n.66‐17 of May 5, 2017).

## Data Availability

Data is contained within the article.
